# Yarrow supercritical extract exerts antitumoral properties by targeting lipid metabolism in pancreatic cancer

**DOI:** 10.1371/journal.pone.0214294

**Published:** 2019-03-26

**Authors:** Lamia Mouhid, Marta Gómez de Cedrón, Elena García-Carrascosa, Guillermo Reglero, Tiziana Fornari, Ana Ramírez de Molina

**Affiliations:** 1 Molecular Oncology and Nutritional Genomics of Cancer, IMDEA-Food Institute, CEI UAM + CSIC, Madrid, Spain; 2 Production and Characterization of Novel Foods Department, Institute of Food Science Research CIAL, CEI UAM + CSIC, Madrid, Spain; Flinders University of South Australia, AUSTRALIA

## Abstract

Metabolic reprogramming is considered a hallmark of cancer. Currently, the altered lipid metabolism in cancer is a topic of interest due to the prominent role of lipids regulating the progression of various types of tumors. Lipids and lipid-derived molecules have been shown to activate growth regulatory pathways and to promote malignancy in pancreatic cancer. In a previous work, we have described the antitumoral properties of Yarrow (*Achillea Millefolium)* CO_2_ supercritical extract (Yarrow SFE) in pancreatic cancer. Herein, we aim to investigate the underlaying molecular mechanisms by which Yarrow SFE induces cytotoxicity in pancreatic cancer cells. Yarrow SFE downregulates SREBF1 and downstream molecular targets of this transcription factor, such as fatty acid synthase (FASN) and stearoyl-CoA desaturase (SCD). Importantly, we demonstrate the *in vivo* effect of Yarrow SFE diminishing the tumor growth in a xenograft mouse model of pancreatic cancer. Our data suggest that Yarrow SFE can be proposed as a complementary adjuvant or nutritional supplement in pancreatic cancer therapy.

## Introduction

Pancreatic cancer is the second leading cause of cancer-related deaths worldwide. It is an aggressive malignancy with poor prognosis: the overall 5-year survival rate is less than 5%. Risk factors described to be linked with its development are obesity and chronic pancreatitis, but also tobacco smoking, heavy alcohol intake, unbalanced diets, or high red meat intake [1,2]. Given the absence of indicators of illness, it is often diagnosed at metastatic late stages. Although surgery remains the main beneficial treatment followed by chemotherapy and radiation, current therapies do not improve patient’s survival. Therefore, the development of effective therapeutic strategies targeting molecular alterations associated with pancreatic tumor growth and the resistance to apoptosis are needed to improve their survival.

In the last years, there has been a growing interest in the use of phytochemicals and dietary-derived compounds for prevention or for cancer treatment [3,4]. Thus, some of them have shown antitumoral properties *in vitro* and *in vivo* [4–6]. In this regard, there are some phytochemicals derived from natural sources, such as taxol and camptothecin, which are extensively used in clinics to treat several tumors [7,8]. Nevertheless, pancreatic cancer remains one of the most resistant tumor [9]. Current chemotherapy is based on the use of gemcitabine, 5-fluorouracil, irinotecan and/or oxaliplatin but new complementary approaches are required.

On the other hand, metabolic reprogramming is well-recognized as a hallmark of cancer [10] and thus, there is an increased interest for targeting its altered metabolism. Tumors display high rates of cell proliferation and they can acquire malignancy associated to stemness and invasive properties. Moreover, highly proliferative cells hold an exacerbated glucose uptake sustaining aerobic glycolysis (Warburg effect) for anabolic processes [11–13]. In addition, increased glutamine uptake and glutaminolysis support carbon and nitrogen backbones for anabolic purposes. Nitrogen, in addition to its role in protein and nucleotide biosynthesis, is crucial for the synthesis of glutathione and essential to maintain the redox homeostasis [12–14].

Cancer cells are also characterized by having an increased *de novo* fatty acid synthesis which contributes to the carcinogenic process and cancer cell survival. Metabolic fatty acid (FA) enzymes are essential for neoplastic growth as well as for the signaling of key tumorigenic pathways. During tumor development, lipid associated alterations include an increase in lipogenic enzymes expression such as fatty acid synthase (FASN), acetyl carboxylase (ACC), stearoyl-CoA desaturase (SCD), ATP citrate lyase (ACLY), and an increase in the synthesis and uptake of cholesterol. As a result, tumor cells can control membrane fluidity, which has an impact on intracellular oncogenic signaling pathways, and sharpens resistance to chemotherapeutics. In addition, the increased storage of lipid and cholesterol molecules into lipid droplets allows tumor cells to gain independence from the extracellular nutrient availability [15,16].

In this context, many authors have described alterations in expression of lipid metabolic genes, and their link with the development and prognosis in cancer [17,18]. Thus, targeting specific enzymes involved in fatty acid and cholesterol synthesis, or affecting genes involved in their transcriptional regulation could be a novel approach for cancer treatment [19,20].

Recently we have described the antitumoral properties of *Achillea Millefolium* derived extract, commonly known as Yarrow, obtained by Supercritical Fluid Technology (SFE), in pancreatic cancer cell lines [21]. Yarrow SFE diminishes cell viability of pancreatic cancer cells in a dose dependent manner, induces apoptosis and inhibits anchorage independent cell growth. Moreover, Yarrow SFE synergizes with 5-fluororacil (5-Fu) which is currently used in the clinics in pancreatic cancer treatment [22]. Herein, we identify Sterol Regulatory Element-Binding Transcription Factor 1 (SREBF1) as a molecular target of Yarrow SFE which is implicated, at least partially, in the antitumoral activities observed. Importantly, downstream molecular targets of SREBP1, such as FASN and SCD, are also diminished both at the transcriptional and post-transcriptional levels. These alterations would affect intracellular lipid homeostasis which also correlate with the inhibition of invasiveness properties of pancreatic cancer cells.

Importantly, we also demonstrate the *in vivo* effect of Yarrow SFE diminishing the tumor growth in a xenograft model of pancreatic cancer. Based on these results, Yarrow supercritical extract could be proposed as a complementary adjuvant in pancreatic cancer therapy.

## Methods

### Yarrow extract

Yarrow SFE was obtained by CO_2_ supercritical fluid extraction (Thar Technology, model SF2000). Briefly, a CO_2_ flux of 70 g/min, 140 bar and 40°C for 180 minutes was applied to 400 g of dried and grounded Yarrow in a 2L cylinder. The resulting extract was collected with absolute ethanol, and the dissolvent was removed using a rotavapor at low temperature (30°C).

The extract contains volatile oils which comprises monoterpenes such as eudesmol (11.19%) and borneol (16.28%) as major compounds, and sesquiterpenes as caryophyllene (4.90%), α-cadinol (8.22%), α-bisabolol (2.73%), among others [21].

### Cell culture

Pancreatic cancer cells MIA PaCa-2 and PANC-1 were purchased from American Type Culture Collection (Manassas, VA) and were cultured in DMEM media (2 mM glutamine) and 10% fetal bovine serum (LONZA Iberica, S.A) in 5% CO_2_ atmosphere at 37°C and 95% humidity. 5 x 10^6^ cells were seeded in M6 multiwell plates for microarray assays and in a p60 culture dishes to isolate proteins for WB analysis and mRNA for qPCR.

### RNA isolation and gene expression assay

MIA Paca-2 and PANC-1 cells were plated and after an overnight incubation, cells were treated with 30 and 70 μg/mL of Yarrow extract for 48 hours. Non-treated cells were kept as controls. Total RNA was isolated from each condition with RNeasy Mini Kit (Qiagen Iberica) following manufacturer´s instructions. RNA quality and quantity were checked by UV spectroscopy (Nanodrop 2000 Spectrophotometer, Thermo Scientific, Waltham).

RNA extracted from MIA PaCa-2 was subsequently used for a comparative microarray gene expression analysis between non-treated and 30 and 70 μg/mL Yarrow treated cells. The analysis was performed at the Genomic Service Facilities at the National Center of Biotechnology (CNB-Madrid, Spain). RNA integrity was first determined using a 2100 Bioanalyzer (Agilent Technologies, Santa Clara, CA). RNAs were then reverse transcribed and fluorescently tagged using the one color Low Input Quick Amp Labeling Kit (Agilent Technologies), according to manufacturer’s instructions. The complementary RNAs were hybridized in an Agilent Sure Print G3 Human 8x60 K (Whole Human Genome Microarray Kit) platform, using the one-color gene expression system as described by the manufacturer (Agilent Technologies).

### Quantitative real-time polymerase chain reaction (qPCR)

Same amounts for each RNAs samples were reverse transcribed by a high Capacity cDNA Archive Kit (applied Biosystems) for 2 hours at 37°C. To determine gene expression, Realtime-qPCRs were performed on the 7900HT-Real-Time PCR System (Applied Biosystems, Foster City, CA) using the TaqMan gene expression assays. The probe sets were Hs01005622, Hs01682761, Hs01088691, Hs00607129, Hs 1597989, Hs99999901_s1 for FASN, SCD, SREBF1, HSPA-5, ASS1 and 18s, respectively. For the isoforms SREBP1-a and SREBP1-c, the primers used to quantify their expression were hSREBP-1a (Fw: GGAGGGGTAGGGCCAACGGCCT; Rv: CATGTCTTCGAAAGTGCAATCC) and hSREBP-1c (Fw: TCAGCGAGGCGGCTTTGGAGCAG; Rv: CATGTCTTCGATGTCGGTCAG). The relative expression quantity for each gene (RQ) was determined following the 2-ΔΔCt Livak method [23].

RQ Manager Software and Expression Suite Software (Applied Biosystems) were used to calculate the relative expression of each gene.

### Intracellular cholesterol and neutral lipid content

To determine the total intracellular cholesterol, the assay was carried out following the instructions of Cholesterol Detection Kit (cell-based) (BioVision Inc, San Francisco, USA). Briefly, cells were cultured with 50 and 70 μg/mL Yarrow for 48 hours before to be extracted with 200 μL of chloroform: Isopropanol: NP-40 (7:11:0.1) in a micro-homogenizer. The lipid fraction was collected and mingled with a cholesterol enzyme mix (which includes cholesterol esterase), and the absorbance of the resultant colorimetric assay was measured at 570 nm.

Quantification of neutral lipid content was done by mean of Bodipy staining. As a brief description, cells were treated with 2 μM BODIPY staining solution (BODIPY 493/503, Invitrogen) in PBS for 15 min at 37°C and fixed with 4% paraformaldehyde (PFA). Images were obtained with a Leica DM IL microscope, 40X Plan Fluotar objective and registered using Leica Application Suite (LAS).

### Immunoblot analysis

After 48 hours incubation with Yarrow SFE, cells were washed and detached using trypsin. Protein lysates were obtained with Laemmli buffer (60 mM Tris-HCl, 10% glycerol 2% SDS) and centrifuged at 5000 rpm for 5 minutes. After quantification (BCA-Piercing), denaturalized protein samples were separated through a 4–15% Mini-Protean TGX Precast Protein Gel (BioRad) and transferred onto a nitrocellulose membrane using Trans-Blot Turbo Transfer System (BioRad). Membranes were incubated in 5% non-fat dried milk PBS (1% Tween-20) at room temperature for 1 hour and then incubated overnight with primary antibodies against FASN (Cell Signaling) and SCD (kindly provided by Dr Demoulin [24]. α-tubulin (Sigma-Aldrich) was used as a loading control.

### Invasion through matrigel

To evaluate the effect of Yarrow SFE on invasiveness, MIA PaCa-2 cells were treated for 48 hours with different doses of Yarrow SFE. After discarding dead cells, a total of 2.2×10^4^ alive ones were then maintained in 1% FBS DMEM (without extract) and seeded onto Matrigel-coated Transwell chambers) (8.0 μm pore) (Corning Life Sciences). In the lower chamber, 700 μL of DMEM with 10% FBS were added as a chemo attractant. 48 hours later, inserts were washed twice with PBS and fixed with 4% paraformaldehyde prior to crystal violet staining. Cells that have invaded through Matrigel in four random fields were counted using a light microscope at ×10 magnification.

### Animals and tumor xenograft model

All procedures were approved by the Animal Care and Use Committee of the Consejo Superior de Investigaciones Científicas (CSIC, Spain), and by the authority of the Comunidad of Madrid (PROEX112/17). Male Hsd: Athymic Nude-Foxn1nu nude mice (7 weeks old, weighing 26–31 g) were purchased from Envigo RMS (Spain). Mice were kept in a temperature and humidity-controlled environment, with 12 hours light/dark-cycle and had access to water and standard chow (Safe, D40 RMM. Proteins 15.2%, Fat: 3.2%, Minerals Ash: 4.4%, Fiber: 4.1%) *ad libitum*.

The xenograft model was obtained by injecting subcutaneously into the back loin 1,5 x10^6^ MIA PaCa-2 cells diluted in DMEM: Matrigel (50:50). After 15 days, 8 animals received Yarrow SFE extract (1000 mg/Kg), diluted in corn oil (Sigma-Aldrich) in 150 μL as final volume. The treatment was administrated using an orogastric silicon-tube, three times per week for 15 days. Intragastrical administration of an equal volume of corn oil alone was given to 8 control animals.

Human endpoints were established if tumors had reached 1.6 cm^3^ or more, or if there had been signs of ulceration, or mice had lost 20% or more of body weight, or if mice had presented piloerection, stereotyped or aggressive behaviors, pain indicative postures, lack of activity and tremors or convulsions. Animals were daily monitored, assessing the room temperature and humidity, and their health by checking their behavior and general appearance. Body weight was measured weekly. The antitumor effect of Yarrow was monitored by estimating tumor volume (mm^3^) and calculated as (W^2^ x L) x 0.52, where W is tumor width (in mm) and L is tumor length (in mm).

Finally, animals were euthanized by cervical dislocation.

### Immunohistochemistry

Immunohistochemistry was performed following standard protocols. Mouse monoclonal antibody against Ki-67 (IR626, DAKO) was used on tissue paraffin sections (4μm). Images were obtained through the Axio Scan Z1 (Zeiss). The quantification of Ki-67 positive cells was done with ZEN Lite 2.3 software (Zeiss).

### Statistical analysis

Microarray gene expression data were obtained and analyzed using FIESTA software (version 1.0, http://bioinfogp.cnb.csic.es/tools/FIESTA; Centro Nacional de Biotecnología, Madrid, Spain). Statistical analyses were determined by the Limma package (linear models for microarray data) (Smyth, 2004), using a p-value<0.05 as limit of significance. Overexpression and repression were considered from a minimum change of 2-fold of the non-treated cells (control).

RT-qPCR results were analyzed by using two-way ANOVA (Bonferroni post hoc test). Western Blot results were quantified by Image J and statistically analyzed by one-way ANOVA (Bonferroni post hoc test). One-way ANOVA was also used to determine differences in invasion and 3D-culture. Results of *in vivo* experiments were analyzed with t-student statistical analysis. Statistical differences were considered significant when p-value was <0,05.

All the statistical analysis was performed with GraphPad Prim 6 statistical software.

## Results

### Yarrow SFE downregulates the expression of the lipid metabolism-master regulator SREBF1

In a previous work we have described that Yarrow SFE inhibits cell viability of MIA PaCa-2 and PANC-1 pancreatic cancer cell lines, with an induction of apoptotic cell death [22].

In order to identify the molecular targets implicated on the mechanism of action of Yarrow SFE, a comparative gene expression microarray (G2519F-026652 Human Gene Expression v2 4x44K Microarray) was performed (link to raw data: https://www.ncbi.nlm.nih.gov/geo/query/acc.cgi?acc=GSE124043). MIA PaCa-2 cells were treated with two different doses of Yarrow (IC_50_: 31,45 ±8,56 μg/mL and 2x IC_50_) for 48 hours. Genes whose expression was significantly altered at both concentrations (p-value<0.05), with a minimum of 2-fold change overexpression or repression, compared to control non-treated cells (DMSO), are listed in **Table 1**.

**Table 1:** Microarray data of differentially expressed genes after treatment of MIA PaCa-2 pancreatic cancer cell line with 30 and 70 μg/mL Yarrow for 48 hours. Data represent the value of the most significant probe for three independent experiments for each condition. Genes with a statistical significant difference (p-value< 0,05) and more than 2-fold absolute change variation compared to control (DMSO) are shown.

Yarrow (μg/mL)	Genes	Fold Change	P-value (Limma)	Systematic Name	Description	Cancer Ref.	Pancreatic Cancer Ref.
30	**ASS1**	-3,62	0,033	NM_000050	Homo sapiens argininosuccinate synthase 1, transcript variant 1	[25–27]	[28–30]
70		-4,38	0,0477				
30	**CHD5**	2,84	0,0036	NM_015557	Homo sapiens chromodomain helicase DNA binding protein 5	[31–33]	[34]
70		2,40	0,0026				
30	**CREBZF**	2,82	0,0017	NM_001039618	Homo sapiens CREB/ATF bZIP transcription factor, transcript variant 1	[35,36]	
70		2,08	0,0214				
30	**CSTF3**	2,08	0,0355	NM_001033505	Homo sapiens cleavage stimulation factor, transcript variant 2	[37,38]	
70		2,08	0,0269				
30	**DHRS3**	-2,78	0,0178	NM_004753	Homo sapiens dehydrogenase /reductase (SDR family) member 3	[39,40]	
70		-2,52	0,0435				
30	**HLA-G**	-2,80	0,0415	NM_002127	Homo sapiens major histocompatibility complex, class I, G	[41–43]	[44,45]
70		-3,58	0,0280				
30	**HSPA5**	-2,44	0,0406	NM_005347	Homo sapiens heat shock 70kDa protein 5 glucose-regulated protein,78kDa	[46–49]	[50–52]
70		-2,51	0,0444				
30	**ICAM5**	-2,05	0,0145	NM_003259	Homo sapiens intercellular adhesion molecule 5, telencephalin	[53,54]	
70		-2,02	0,0196				
30	**ING3**	2,44	0,0035	NM_019071	Homo sapiens inhibitor of growth family, member 3 transcript variant 1	[55,56]	
70		2,03	0,0152				
30	**MEF2D**	-2,98	0,0144	NM_005920	Homo sapiens myocyte enhancer factor 2D, transcript variant 1	[57–59]	
70		-2,18	0,0155				
30	**PALM**	-3,21	0,0028	NM_002579	Homo sapiens paralemmin, transcript variant 1	[60,61]	
70		-2,09	0,0271				
30	**RAB11FIP1**	-2,06	0,0334	NM_001002814	Homo sapiens RAB11 family interacting protein 1 (class I), transcript variant 3	[62]	
70		-2,11	0,0448				
30	**SERPINA3**	-3,01	0,0078	NM_001085	Homo sapiens serpin peptidase inhibitor, clade A (alpha-1 antiproteinase, antitrypsin), member 3	[63,64]	
70		-2,27	0,0061				
30	**SHC1**	-2,21	0,0237	NM_003029	Homo sapiens SHC (Src homology 2 domain containing) transforming protein 1, transcript variant 2	[65]	[66]
70		-2,01	0,0359				
30	**SKAP2**	-2,61	0,0149	ENST00000345317	Src kinase associated phosphoprotein 2	[67,68]	[69]
70		-2,08	0,0346				
30	**SREBF1**	-2,75	0,0004	NM_001005291	Homo sapiens sterol regulatory element binding transcription factor 1, transcript variant 1	[70–74]	[75,76]
70		-2,05	0,0064				
30	**TRIM44**	-2,13	0,0351	NM_017583	Homo sapiens tripartite motif containing 44	[77–79]	
70		-2,07	0,0371				

**Fig 1:**
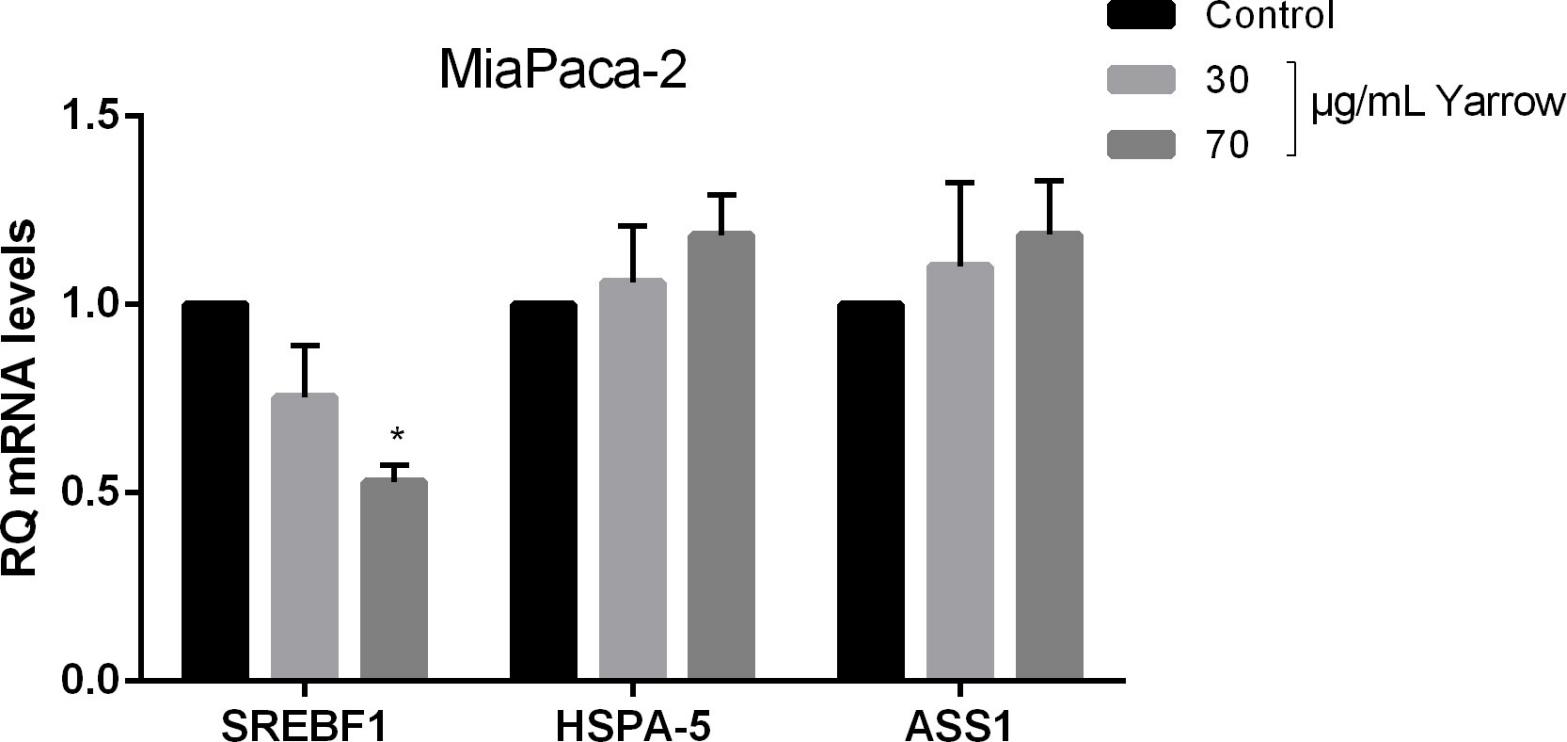
Microarray validation. mRNA relative expression in human pancreatic cancer-derived cells (MIA PaCa-2) treated with two different concentrations of Yarrow extract (30 and 70 μg/mL) in comparison with non-treated cells (subtracting the endogenous control 18S ribosomal RNA). Data represent the mean ± S.E.M of three independent experiments each one performed in triplicate. Asterisks indicate statistical differences in treated cells in comparison with the control (DMSO) *p<0.05.

SREBF1 (Sterol Regulatory Element Binding Transcription Factor), ASS1 (Argininosuccinate Synthase) and HSPA-5 (Homo Sapiens Heat Shock protein 5) were selected for further validation by RT-qPCR due to their known association with tumor processes and with pancreatic cancer development. As shown in **Fig 1**, Yarrow SFE significantly diminished the expression levels of SREBF1 in a 25% and 48% after 30 and 70 μg/m treatments respectively, compared to control cells (DMSO).

Sterol regulatory element binding proteins (SREBP) are master transcriptional regulators of the synthesis and uptake of lipids and cholesterol [80]. In mammals, there are two isoforms of SREBP proteins, SREBP1 and SREBP2. SREBP1 is mainly implicated in the expression of genes for *de novo* synthesis of fatty acids, meanwhile SREBP2 is dedicated to the regulation of genes involved in the synthesis and uptake of cholesterol. Nevertheless, SREBP1 and 2 significantly overlap in the regulation of lipid homeostasis [81,82].

SREBP1 has been found highly upregulated in several cancers. In humans, it includes, in turn, two isoforms: SREBP1c which regulates FA metabolism, and SREBP1a which is implicated in both fatty acid (FA) and cholesterol metabolism [81].

**Fig 2:**
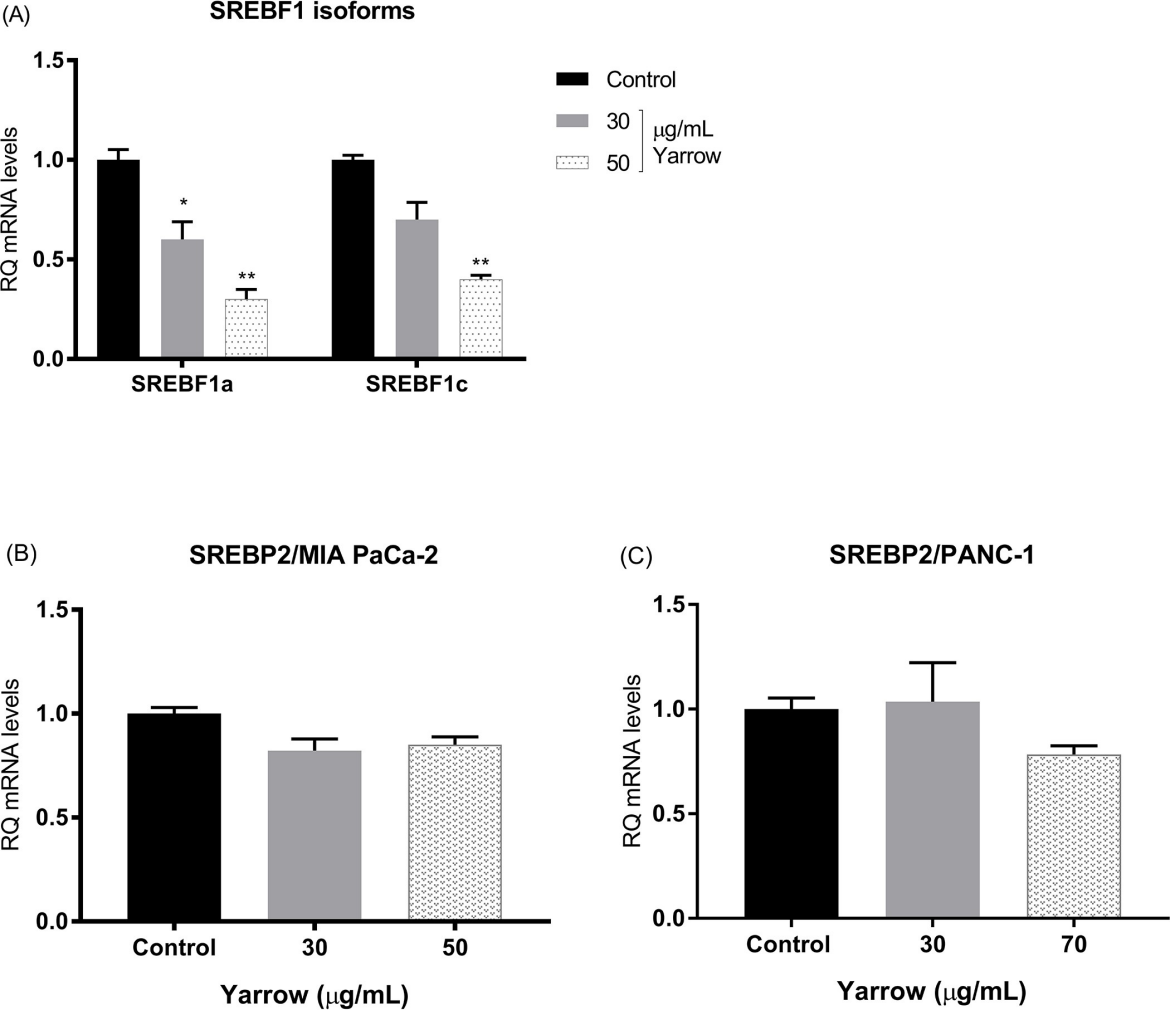
Yarrow SFE inhibits the expression of SREBP1 but not the expression of SREBP2. (A) Expression levels of SREBF1a and SREBF1c isoforms, (B) and levels of SREBF2 mRNA relative expression in human pancreatic cancer-derived MIA PaCa-2 (C) and PANC-1 cells treated 48 hours with different concentrations of Yarrow SFE. Data represent the mean ± S.E.M of three independent experiments each one performed in triplicate. Asterisks indicate statistical differences in treated cells in comparison with control (DMSO) and *p<0.05; **p<0.01.

As individual functions of SREBPs are overlapped, we wanted to determine the effect of Yarrow SFE on the expression of the two SREBP1 isoforms. As shown in **Fig 2A**, RT-qPCR analysis showed that Yarrow SFE diminished the expression of SREBF1a and 1c similarly in a dose-dependent manner.

On the other hand, the altered cholesterol homeostasis is frequently linked to the tumorigenic process and malignancy of tumors [83]. Considering that SREBP1a and SREBP2 share around 45% of homology at the protein level, we wonder if Yarrow SFE extract could affect the expression of SREBF2 in a similar manner it does with SREBF1. Treatments with Yarrow SFE did not show a significant affectation of the expression levels of SREBF2 neither in MIA PaCa-2 (**Fig 2B**) nor in PANC-1 (**Fig 2C**). Indeed, when we analyzed the intracellular cholesterol content, we did not found differences between Yarrow SFE treatments compared to control cells (**S1 Fig**), certainly due to the non-alteration of SREBP2 expression.

These results suggest that SREBP2 can maintain the cholesterol homeostasis in spite of the downregulation of SREBP1, where Yarrow SFE specifically targets the expression of SREBF1 transcription factor.

Importantly, as the activity of SREBP1 is low in normal tissues and aberrantly increased in cancer pathogenesis [84], its inhibition has been considered a promising strategy for cancer treatment [75,82].

### Yarrow SFE downregulates FASN and SCD downstream molecular targets of SREBP1

Multiple studies have revealed that oncogenic pathways such as PI3K/Akt signaling, which regulates glucose metabolism, and oncogenic Myc, which regulates glutamine metabolism, end up with the upregulation of SREBP1 to promote fatty acid synthesis [84–86].

**Fig 3:**
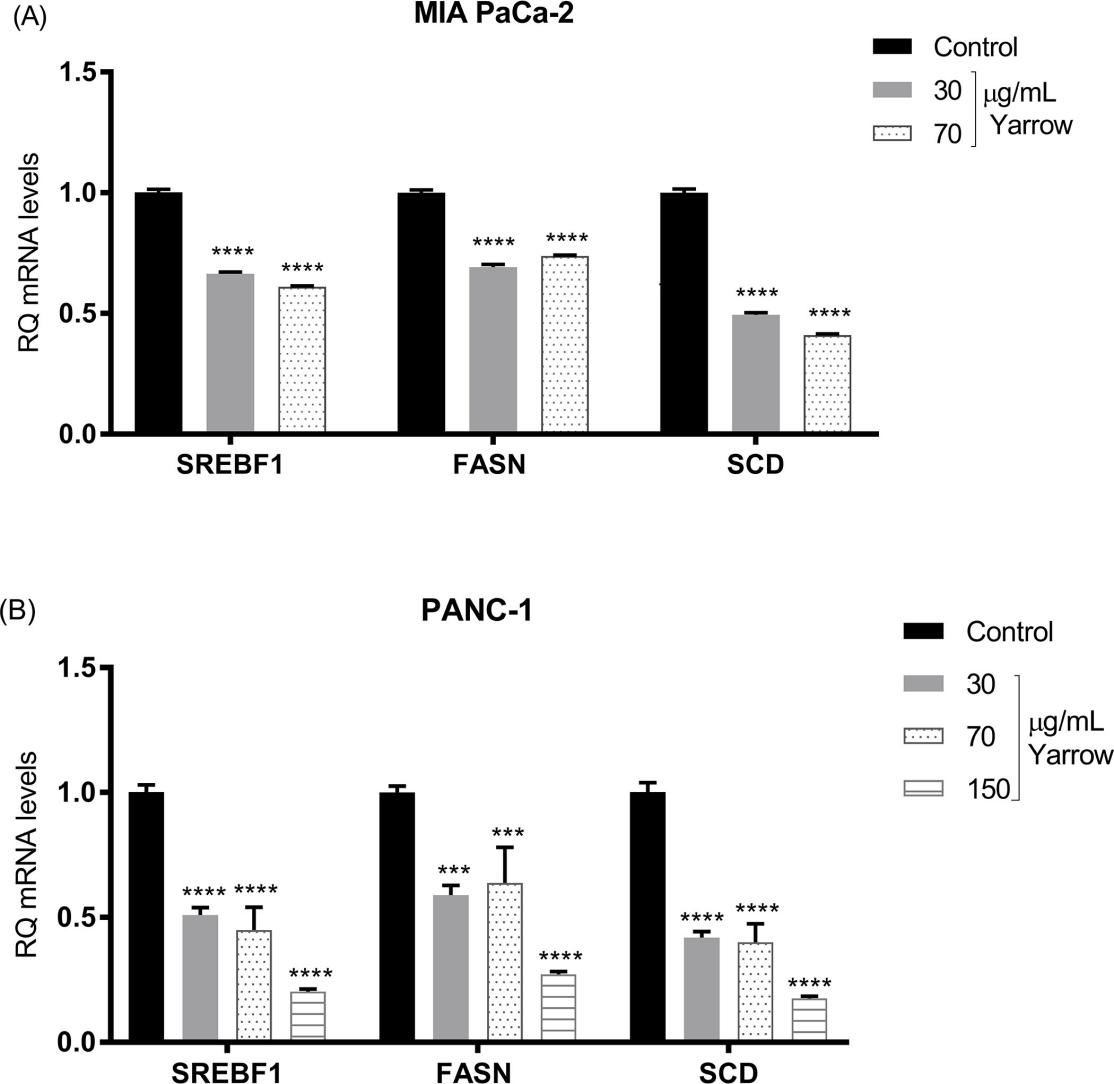
Yarrow SFE inhibits the expression levels of SREBP1 molecular targets FASN and SCD in MIA PaCa-2 and PANC-1 pancreatic cancer cells. (A) mRNA relative expression in human pancreatic cancer-derived MIA PaCa-2 and (B) PANC-1 cells treated 48 hours with different concentrations of Yarrow extract. Data represent the mean ± S.E.M of three independent experiments each one performed in triplicate. Asterisks indicate statistical differences in treated cells compared to control (DMSO) and ***p<0.001; ****p<0.0001.

**Fig 4:**
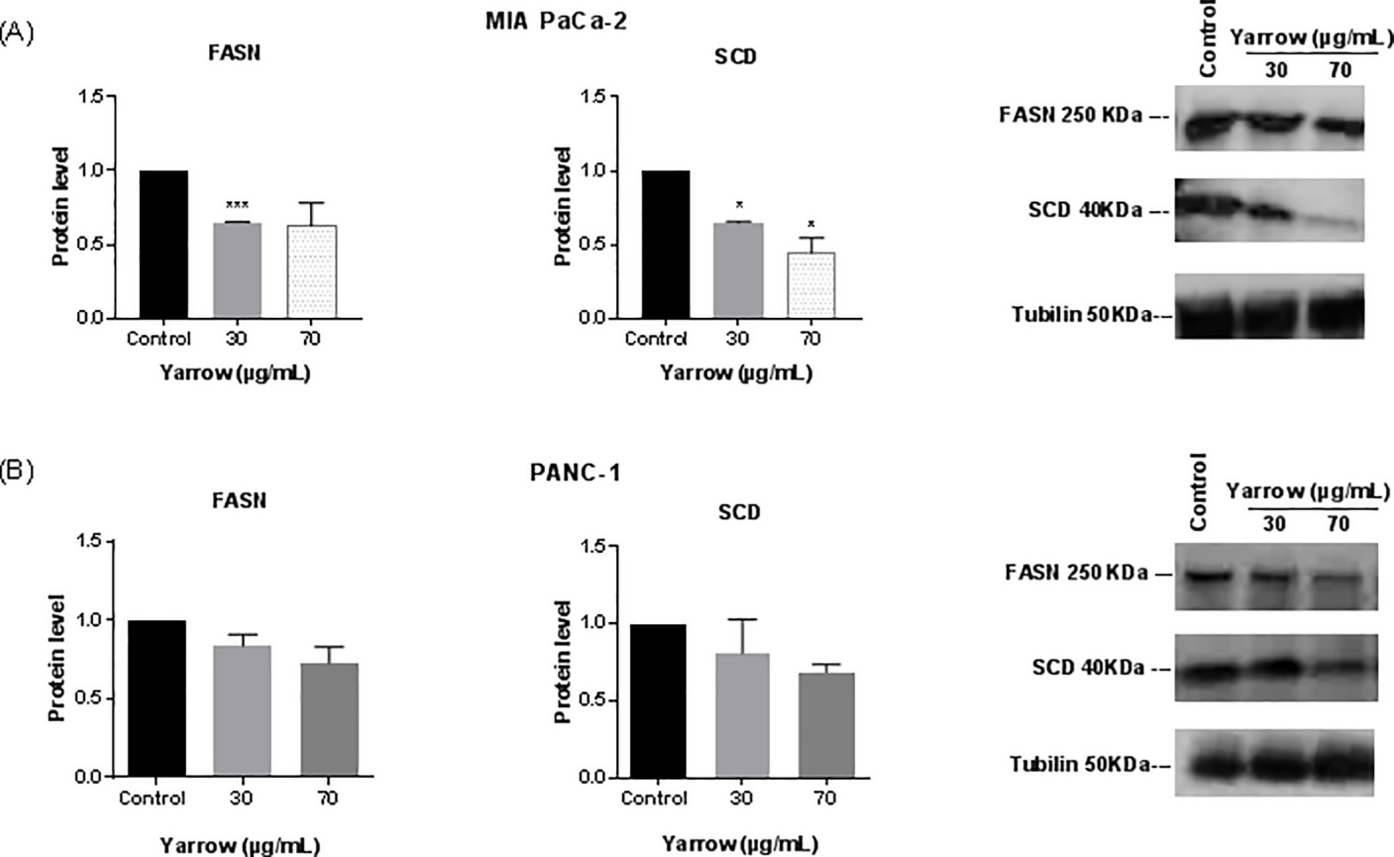
Yarrow SFE extract diminishes FASN and SCD protein levels in MIA PaCa-2 and PANC-1 pancreatic cancer cels. (A) Western blot protein analysis and quantification of FASN and SCD in MIA PaCa-2 and (B) in PANC-1 cell lines treated with Yarrow SFE extracts for 48 hours. Bar data represents the mean ± S.E.M of three independent experiments each one performed in duplicate, and bands are representative of one experiment. Asterisks indicate statistical differences in treated compared to non-treated cells *p<0.05; ***p<0.001.

Thus, SREBP1 connects oncogenic upregulation of glycolysis and/or glutaminolysis to lipogenesis [11], by inducing the expression of Fatty Acid Synthase (FASN) and the Stearoyl-CoA desaturase (SCD), among others. As we did not have observed any significant difference regardless cholesterol levels, we wonder if lipogenic genes regulated by SREBP1, such as FASN and SCD, could be altered. Yarrow SFE treatments of MIA PaCa-2 cells for 48 hours downregulated the expression levels of FASN and SCD enzymes, both at the transcriptional (**Fig 3A**) and at the post-transcriptional levels (**Fig 4A**).

We obtained similar results after Yarrow SFE treatment in PANC-1, a pancreatic cancer cell line which has been described to be more chemo and radio-resistant, compared to MIA PaCa-2 cells [87,88]. **Fig 3B** shows a significant downregulation of the mRNA expression levels of SREBP1, FASN and SCD in PANC-1 pancreatic cancer cell line, and **Fig 4B** shows a tendency towards a reduction in the protein expression levels of FASN and SCD.

The effect of Yarrow SFE on SREBF1 downregulation seems to be highly specific, as similar results were obtained in other cancer types, such as in SW-620, a colon cancer cell line (**S2 Fig**).

### Yarrow SFE diminishes intracellular lipid content and cell invasion

**Fig 5:**
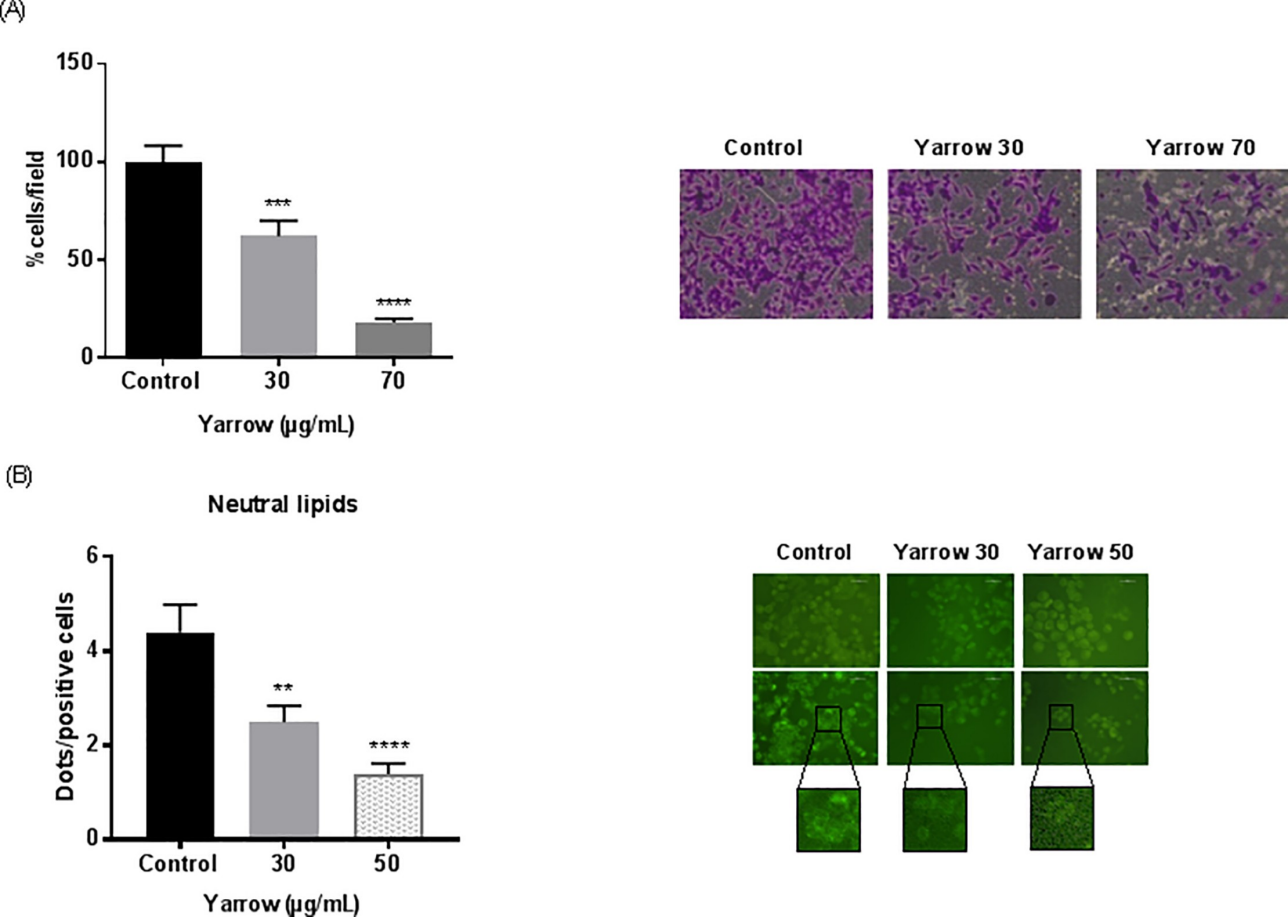
Yarrow SFE extract reduces MIA PaCa-2 cell invasion and intracellular neutral lipid content. (A) Quantification of cells invading through Matrigel-coated chambers after 48h of treatment with Yarrow SFE compared to non-treated control cells (DMSO). Data represent mean ± S.E.M of three independent experiments each one performed in duplicate. (B) Representative immunofluorescence images of the fluorophore Bodipy distribution in MIA PaCa-2 treated cells with Yarrow SFE and non-treated cells, carried out twice. Asterisks indicate statistical differences between Yarrow SFE treated cells compared to non-treated cells (**p<0,01; ***p<0.001; ****p<0.001).

Tumor cell invasion is an important read-out of the aggressiveness and metastatic potential of cancer cells. In this regard, it has been described a link between FASN downregulation and the reduction of invasion in cancer [84,89]. Thus, next we aimed to investigate the ability of Yarrow SFE to affect the invasion capabilities of MIA PaCa-2 cells through Matrigel-coated chambers. After 48 hours of exposition to Yarrow SFE extract, cells which still alive were placed onto a Matrigel chamber and those invading the matrix towards the lower chamber were monitored. Yarrow SFE treatment significantly reduced the number of cells that passed through Matrigel in a dose-dependent manner, with a reduction of the invading cells of a 50% and 80% after treatment with 30 and 70 μg/mL of the extract, respectively, compared to control non-treated cells (DMSO) (**Fig 5A**). To further assess the impact of Yarrow SFE in intracellular lipid metabolism, we used the Bodipy fluorophore, a specific dye for cellular neutral lipid droplets content. As shown in **Fig 5B**, MiaPaca-2 treated cells with Yarrow SFE were less prone to neutral lipid droplets accumulation compared to control non-treated cells (DMSO). These results suggest that alterations in lipid homeostasis impact on signaling pathways implicated in invasion.

### Yarrow-SFE inhibits the growth of pancreatic tumor xenografts in athymic nude mice

As we have previously shown *in vitro*, antitumoral effects of Yarrow SFE on pancreatic cancer cells reduce cell viability, inhibit tridimensional cell growth, and induce apoptosis [22]. As here we found an inhibition of invasion and a reduced expression of molecular targets implicated in FA and cholesterol homeostasis, we aimed to investigate whether Yarrow SFE could inhibit *in vivo* the tumor growth of pancreatic cancer cells using a xenograft mouse model.

**Fig 6:**
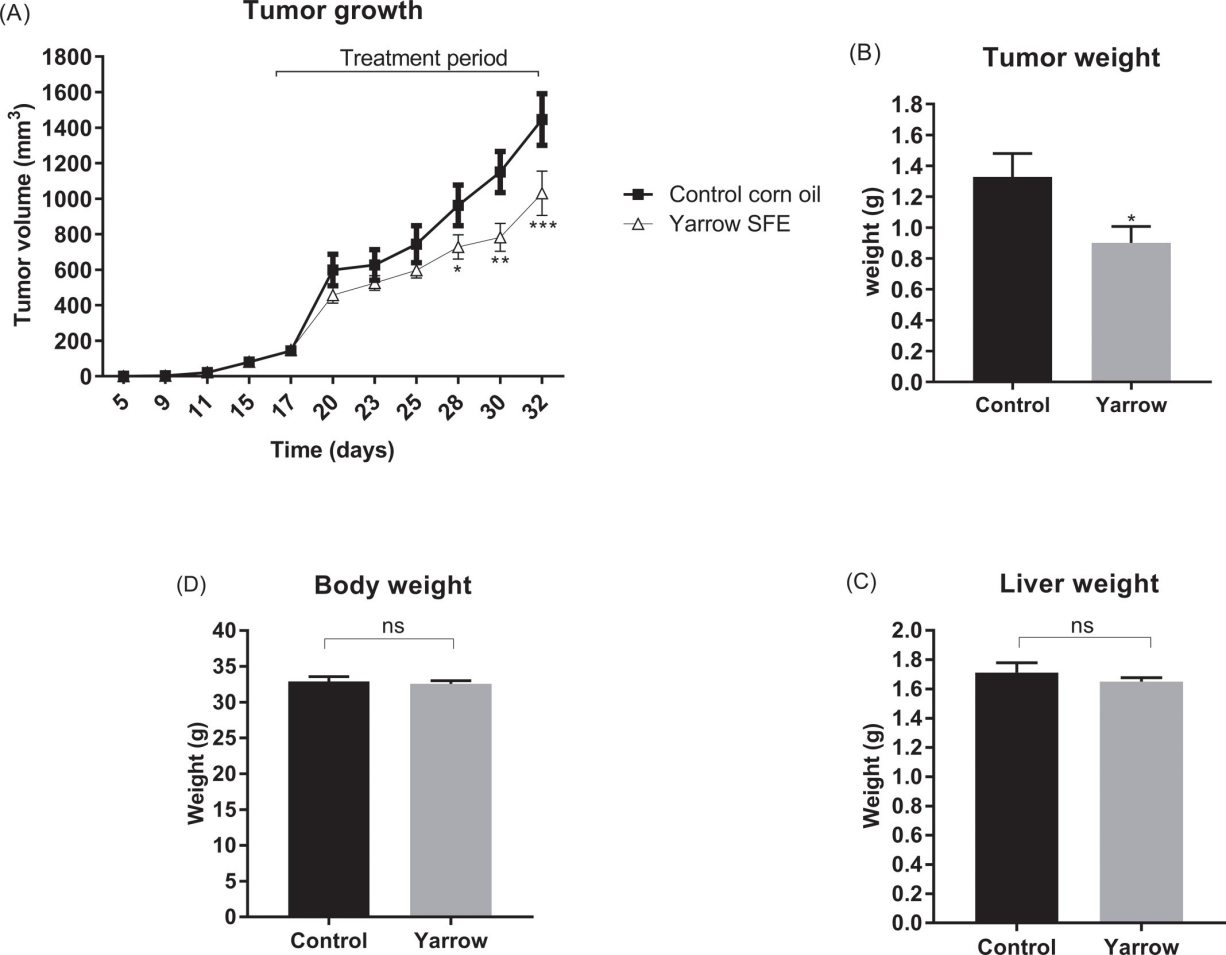
Yarrow SFE diminishes the growth of MIA PaCa-2 tumor xenograft in nude mice. (A) Tumor volume evolution of MIA PaCa-2 xenografts treated with Yarrow SFE extract compared to non-treated control mice (corn oil). (B) Tumor weight, (C) Liver weight and (D) Total body weight quantification at the end of the experiment. Data represents the mean ± S.E.M for 8 treated animals and 8 controls. Asterisks indicate statistical differences in treated with respect to non-treated mice *p<0.05; **p<0.01; ***p<0.001.

With this purpose, we injected 1,5x10^6^ of MIA PaCa-2 cells subcutaneously in nude mice. Tumor volume was measured three times per week from the day of inoculation. When tumors reached an average volume of 100 mm^3^, mice were treated with 1000 mg/kg Yarrow 3 times per week. As shown in **Fig 6A**, tumor growth was reduced in Yarrow SFE treated animals compared to control animals (corn oil) being statistically significant after 10 days of treatment.

Tumor weights at the end of the experiment showed significant reduced values in Yarrow SFE treated mice compared to control animals (Fig 6B). Importantly, we did not find differences regardless body weights nor liver weights (Fig 6C and 6D).

**Fig 7:**
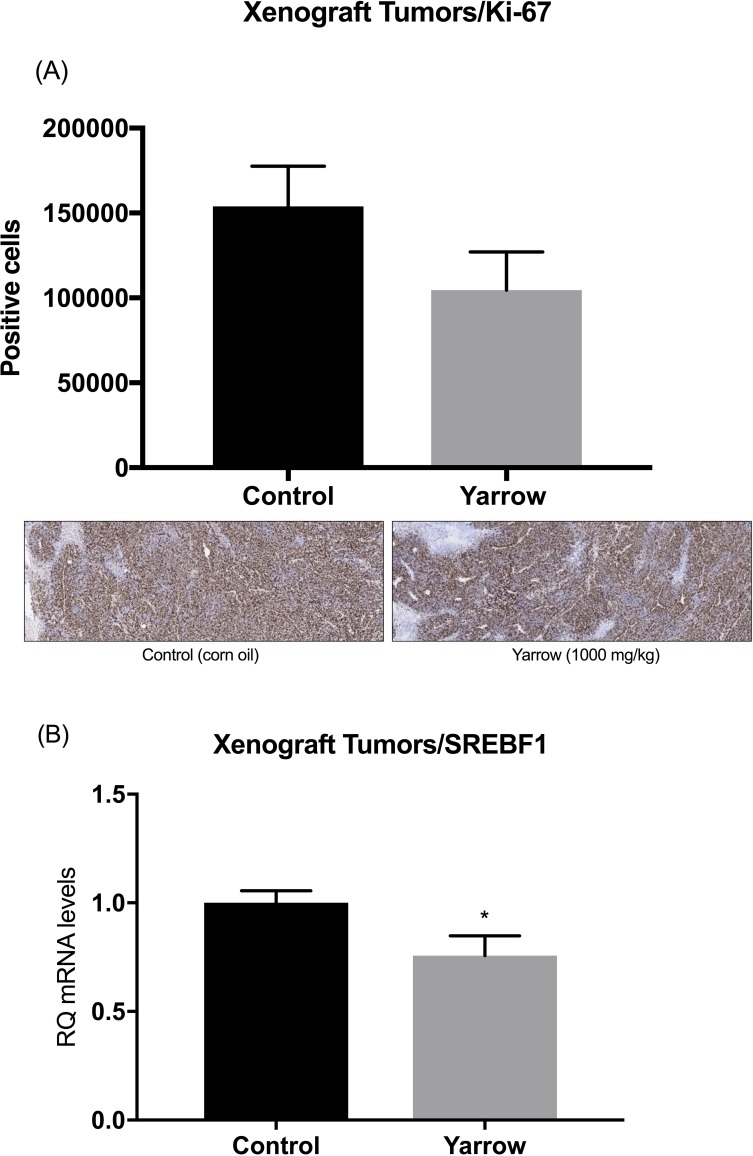
Yarrow SFE diminishes pancreatic cancer cell proliferation in tumor xenografts and SREBF1 expression. (A) Representative images of Ki-67 immunostaining and Ki-67 quantification at the end of the experiment. (B) SREBF1 expression levels in tumors of Yarrow SFE treated mice compared to non-treated control mice (corn oil). Data represent the mean ± S.E.M of five animals for the immunostaining and 8 animals for qPCR. Asterisks indicate statistical differences in treated animals comparing with the control (corn oil) and *p<0.05.

At the end of the experiment, Yarrow SFE treated mice presented a tumor volume reduction of 28.69% (P < 0.05) compared to the control ones (corn oil). Ki-67 staining showed a decrease in the number of Ki-67 positive cells in the tumors of Yarrow SFE treated mice compared to control ones, indicating a reduction in proliferation of tumor cells (**Fig 7A**). Importantly, the expression levels of SREBF1 in the tumors of Yarrow SFE treated animals was also significantly reduced compared to control animals **(Fig 7B**).

## Discussion

Pancreatic cancer remains one of the most uncontrollable and devastating malignant tumors due to its late diagnosis and chemo-refractory profile.

The altered metabolism in tumoral cells has been highlighted in the last years, being an important part of the metabolic reprogramming found in cancer. Lipids, in addition to their role as fuels for ATP production, they play a key role as components of plasmatic and organelles membranes, affecting their plasticity, and are also important as cell signaling molecules. Lipid metabolism alterations have been related to an increase in tumor progression and malignancy. In this regard, SREBP proteins, which are master regulators of lipid metabolism, together with FASN and SCD have been found to be upregulated in several neoplasia. Regardless pancreatic cancer, alterations in lipid metabolism have been also described, such as the increase of *de novo* lipogenesis, which supports tumor growth and malignancy [75,84] and correlates with poorer prognosis in clinics [90].

In this scenario, new approaches to target the altered lipid metabolism in cancer are required to improve cancer patient´s survival. Plant-derived products are a source of bioactive compounds with a reduced toxicity that have great potential in the treatment of chronic diseases conditions including obesity, metabolic syndrome, type 2 diabetes, cardiovascular diseases and cancer [91,92]. Conventional strategies of pharmaceutical industries are focused on isolating pure components as a treatment approach, but plant derived extracts are of great interest because they are composed by several bioactive phytochemicals, as well as because they can act synergistically and target several genes.

In this context, we recently have investigated the antitumoral properties of Yarrow SFE extract (obtained by supercritical fluid extraction -SFE-). Yarrow SFE diminished cell viability of pancreatic cancer cell lines by induction of apoptosis. Moreover, it reduced the ability of these cells to growth and to form colonies in Matrigel. Importantly, Yarrow SFE extract synergized with the antimetabolite 5-fluororacil which is currently used in clinics to treat pancreatic tumors, making the treatment more effective [22].

Herein, by microarray gene expression analysis of MIA PaCa-2 cells treated with Yarrow SFE (at 30 and 70 μg/mL), we identify SREBF1 transcription factor as a direct molecular target of the extract. The same tendency is found in another pancreatic cancer cell line, PANC-1, which is has been reported to be a more aggressive [93]. Yarrow SFE targets similarly SREBF1a and SREBF1c isoforms, but do not significantly downregulates the expression of SREBF2.

SREBP1c binds to the promoter of genes such as FASN (Fatty Acid Synthase), SCD (Stearoyl-CoA Desaturase) or ACC (Acetyl-CoA carboxylase) [94–96], which are essential players for *de novo* synthesis of fatty acids or lipogenesis.

In pancreatic cancer, it has been shown that SREBP1 expression is significantly higher in tumoral tissue compared to adjacent normal tissue. In addition, the aberrant expression of this gene predicts worst prognosis [75]. SREBP1 levels are also increased in other types of cancer, such as prostate, liver, or endometrial tumors, conferring them higher invasion and metastatic capacities [70,73,74]. Thus, SREBP1 has been proposed by several authors as a potential therapeutic target in cancer treatment [82]. In pancreatic cancer cells (PANC-1, PxPC3 and SW1990), SREBP1 silencing reduced proliferation and colony formation. Moreover, SREBF1 silencing diminished the expression of key growth- and apoptosis-regulating genes such as FASN, SCD, TP53, ACC, that results in the decrease of intracellular lipid content and apoptosis [97]. These results are similar to ours after Yarrow SFE treatments, where there is a reduction of SREBF1 expression levels together with an inhibition of invasion and induction of apoptosis (caspase activation). In addition, we have previously shown that Yarrow SFE is more selective against cancer cell lines than normal cells [22]. As cancer cells present higher expression of SREBF1 than normal cells [75], the observed effect of Yarrow SFE on SREBF1 could explain the specificity against tumor cells, being normal cells more protected.

In patients, together with SREBP1, FASN and SCD are also considered worse prognosis markers. These genes are also diminished in MIA PaCa-2 cells after Yarrow SFE treatments.

In the same way, FASN has been shown to be upregulated in several cancers [16,84], including pancreatic cancer [98,99]. High serum levels of FASN are associated with aggressiveness [98,100] and resistance to the chemotherapeutic gemcitabine [101,102].

Conversely, FASN inhibition have been shown to decrease tumor growth and to drive apoptotic cell death. These cytotoxic and cytostatic effects have been validated in ovarian, prostate and pancreatic cancers [98,103,104]. In addition, FASN pharmacological blockage also synergizes with 5-fluororacil [105] to enhance apoptosis activation [76,106]. In this sense, our results match with previous ones, where Yarrow SFE extract could act as a FASN inhibitor.

Finally, regarding SCD, which catalyzes the rate limiting step in the production of monounsaturated fatty acids (MUFAs), whose levels are directly associated with replication rates in cancer cells [107]. High levels of SCD have been described to increase cell proliferation and survival in pancreatic tumors [107,108], and its inhibition has been shown to brake tumor growth in lung, colorectal, osteosarcoma and breast tumors [65–67]. Thus, being considered as a target for chemotherapy, SCD therapeutic inhibition affects tumor growth in several xenograft models (prostate, kidney, gastric or ovarian tumor [68,69].

In the context of the use of phytochemicals for cancer treatment through a metabolic approach, genistein and ginkgolic acid have already been demonstrated to induce cytotoxicity by downregulating of SREBP1 [71,109]. Promising results have been obtained by luteolin, which also targets FASN [76].

Herein, we demonstrate that Yarrow SFE inhibits SREBF1, FASN and SCD leading to less accumulation of lipid droplets which also correlates with the inhibition of cell invasion. Further studies are needed to better understand how Yarrow SFE could disrupt cell metabolism and/or cell bioenergy through metabolomic approaches and cell bioenergetics flux analysis.

Importantly, we demonstrate the *in vivo* effect of Yarrow SFE inhibiting the tumor growth of xenografts models, which seems to be mediated, at least partially, by the downregulation of SREBP1.

In this sense, our data suggest that Yarrow SFE can be proposed as a complementary adjuvant or nutritional complement in pancreatic cancer therapy.

## Conclusions

Yarrow SFE decreases pancreatic cancer cell proliferation and invasion. We identify SREBF1 together with FASN and SCD, as molecular targets of Yarrow SFE. We also demonstrate the *in vivo* effect of Yarrow SFE, which brakes tumor growth in a xenograft mouse model of pancreatic cancer, together with SREBF1 downregulation in the injected tumors. Our data suggest that Yarrow SFE can be proposed as a complementary adjuvant or nutritional supplement in pancreatic cancer therapy.

## Supporting information

S1 Fig Intracellular cholesterol levels. Relative intracellular cholesterol levels in human pancreatic cancer-derived MIA PaCa-2 (A) cells treated 48 hours two with different concentrations of Yarrow SFE extract. Data represent the mean ± S.E.M of three independent experiments each one performed in triplicate.
(TIF)

S2 Fig Yarrow inhibits the expression of *SREBF1* in SW620 colon cancer cells. Line. mRNA relative expression of SREBF1 in SW620 cells treated 48 hours with different concentrations of Yarrow extract compared to non-treated control cells (DMSO). Data represent the mean ± S.E.M of three independent experiment each one performed in triplicate. Asterisks indicate statistical differences in treated cells with respect to the control (non-treated cells) and *p<0.05**p<0.01; ***p<0.001.
(TIF)

## Abbreviations

SFE: Supercritical Fluid Extraction
SREBF: Sterol Regulatory Element Binding Transcription Factor
FASN: Fatty Acid Synthase
SCD: Stearoyl-CoA Desaturase
5-fu: 5-fluororacil
