# Supplementary figures and images for: Yarrow supercritical extract exerts antitumoral properties by targeting lipid metabolism in pancreatic cancer
Source: PLoS One. 2019 Mar 26;14(3):e0214294. doi: 10.1371/journal.pone.0214294 (PMC6435158; doi:10.1371/journal.pone.0214294)

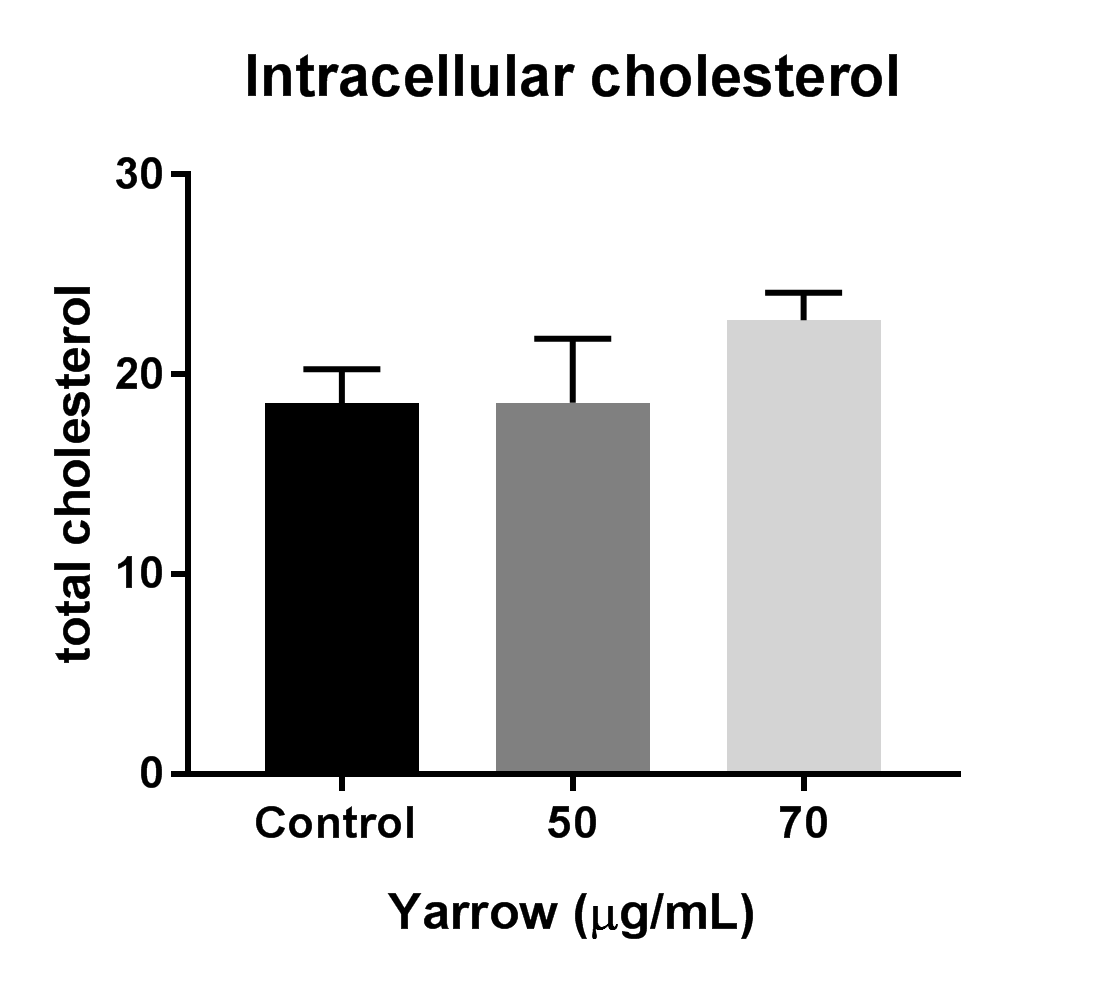

Supplement: S1 Fig — Relative intracellular cholesterol levels in human pancreatic cancer-derived MIA PaCa-2 (A) cells treated 48 hours two with different concentrations of Yarrow SFE extract. Data represent the mean ± S.E.M of three independent experiments each one performed in triplicate. (TIF) [file pone.0214294.s001.tif]

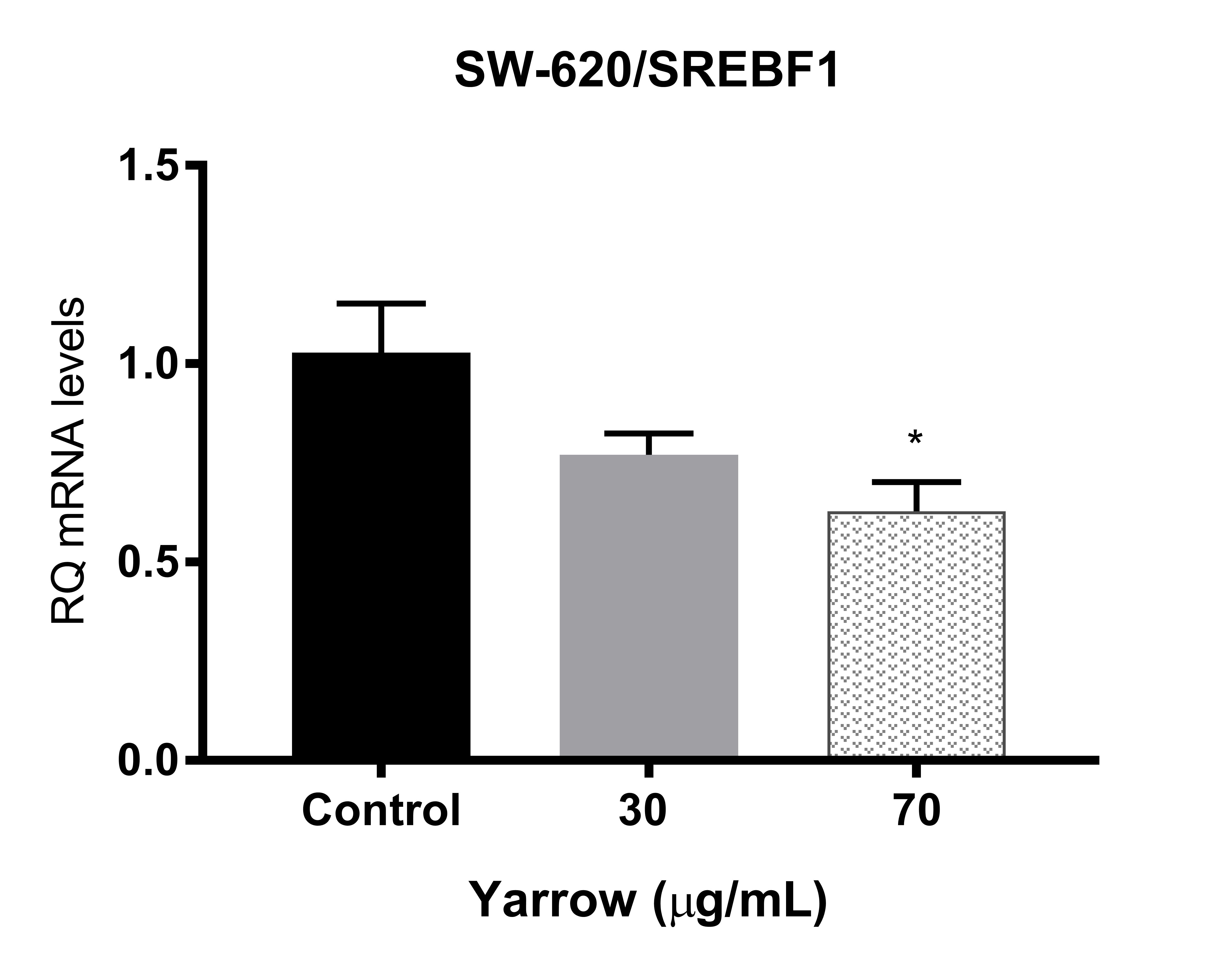

Supplement: S2 Fig — Line. mRNA relative expression of SREBF1 in SW620 cells treated 48 hours with different concentrations of Yarrow extract compared to non-treated control cells (DMSO). Data represent the mean ± S.E.M of three independent experiment each one performed in triplicate. Asterisks indicate statistical differences in treated cells with respect to the control (non-treated cells) and *p<0.05**p<0.01; ***p<0.001. (TIF) [file pone.0214294.s002.tif]
